# Spirituality and quality of life in older adults: a path analysis model

**DOI:** 10.1186/s12877-020-01646-0

**Published:** 2020-07-29

**Authors:** Sara Lima, Lurdes Teixeira, Raquel Esteves, Fátima Ribeiro, Fernanda Pereira, Ana Teixeira, Clarisse Magalhães

**Affiliations:** 1 CESPU, Institute of Research and Advanced Training in Health Sciences and Technologies, Rua Central de Gandra, 1317, 4585-116 Gandra, PRD Portugal; 2 UCIBIO/REQUIMTE, MedTech-Laboratory of Pharmaceutical Technology, Department of Drug Sciences, Faculty of Pharmacy, University of Porto, Porto, Portugal

**Keywords:** Quality of life, Social support, Functionality, Spirituality

## Abstract

**Background:**

Study older adults’ quality of life is becoming increasingly important in the assessment, quality improvement and allocation of health and social care service. The purpose of this study was to enhance knowledge on the relationship between modifiable (psychological variables) and non-modifiable variables (sociodemographic), and quality of life in elderly, regarding psychological and social variables in Portuguese context.

**Methods:**

This is a cross-sectional study, including 604 older adults from general community. 63.6% of the sample was composed by female gender with a mean age of 71.6(SD = 4.81). Participants completed the following instruments: Barthel Index to assess functionality; Satisfaction with Social Support Scale to assess social support; The Spiritual and Religious Attitudes in Dealing with Illness to assess spirituality and Short Form Health Survey 36, to assess mental and physical quality of life.

**Results:**

A path analysis model was performed where the presence of a chronic disease, age and functionality has a direct effect on physical quality of life and spirituality had a direct effect on mental quality of life. Social support mediated the relationship between functionality and mental quality of life, and in turn, functionality mediated the relationship between age and physical quality of life.

**Conclusions:**

Results reinforce the effect of age and chronic disease as non-modifiable variables as well as functionality, spirituality and satisfaction with social support as modifiable variables, in the quality of life of older people. Social support, health and education programs in the community should be promoted in order to improve quality of life in this population. Strategies to promote functionality and enhance the social support network, especially in the elder with chronic illness, should be a priority.

## Background

The introduction of the concept of quality of life (QoL) as a measure of health outcome emerged in the seventies, in the context of medical progress. The increasing prevalence of chronic diseases, with a high incidence in the elderly population, required a focus on the effectiveness of clinical interventions (therapeutic interventions) but also on patient well-being [1].

World Health Organization (WHO), defined QoL as “*an individual’s perception of their position in life in the context of the culture and value systems in which they live, and in relation to their goals, expectations, standards and concerns*” [2] (WHOQOL Group 1994). This definition includes six major domains: (1) physical health, (2) psychological status, (3) levels of independence, (4) social relationships, (5) environmental characteristics, and (6) spiritual standards. Therefore, QoL depends on intrinsic and extrinsic factors, varying from individuals and being subject to influences of their daily life, environment, habits and lifestyle. In order to have a good QoL, individuals` need to function well, fulfilling their role and social functions in a satisfactory way [3].

The dimension of spirituality is fundamental to give meaning to life, to deal with adversity and the experience of the disease, and in ageing people, simultaneously with the normal physical modifications. Regarding these variables, several scientific papers reports in benefits for well-being and quality of life, particularly for the elderly [4] considering the intervention of spirituality dimensions. Historically, Portugal is a mainly Catholic country [5], it has been presenting an increase of those who claim to be believers without religion [6] assuming their individual spirituality understood as the relationship with the transcendent.

Of all age groups, the elderly population is the one with the greatest religious affiliation, asserting itself mostly as a practicing believer. This reality attaches greater importance to the role of religion in the aging process and the management of chronic disease.

Spirituality and religiosity are a resource used for several patients to cope with chronical disease which have a positive impact on their quality of life and well-being [7–9].

The concept of spirituality is extremely general and multidimensional [7, 10, 11] anchored in subjective reasons and influenced by each person’s life experiences, and includes a set of beliefs, that are not associated with religious doctrines [4] in order to disclose a meaning and understanding life search and trust in a transcendent source, in nature or others, with God or a higher power [7, 8]. In religious people, spirituality may reflect the religious doctrine, creeds and philosophical beliefs being the life and disease understands in those perspective [12, 13]. In the literature, religiosity is conceptualised as religious practices, the creeds, beliefs that are reflected in individual’s behaviour, values and way of living [9, 10] that contributes for quality of life in older people [13–15]. It seems that over the years, people tend to give a sense to their life supported in spirituality dimensions and this is also a continuing and evolving stage, giving different importance to the spirituality dimensions according to their meaning in different life events.

Several studies have shown the relationship between spirituality, quality of life and mental health [9, 12, 13, 16]. Although the results cannot be applied to all social groups of the elderly, but there is relative scientific support for these relationship in order to improve the quality of life [13, 17], promote social exchanges and social life, give meaning to life, help face the finitude of life and promote a positive attitude towards aging [18–20]. The belief in the transcendent or in the divine represents an important dimension for the quality of life older people, contributing to the meaning of life, to resilience to difficulties, acting positively as a factor of health promotion [9, 14, 15].

There is a consensus that social support is a multidimensional concept where different dimensions have different impact on individuals. The perception of satisfaction with social support reflects on elderly` health [21] and it is an important indicator of health-related quality of life [16, 19]. Older adults with lower social support reported lower health status being social support a strong predictor of mortality in elderly [20]. According the literature, in elderly people perception of satisfaction with social support was a positive relationship with quality of life [19, 22] being also, predictor variable of quality of life [23, 24]. Supported on above descriptions related with scientific evidences and in order to improve the quality of life in elderly people, the knowledge of the relationships of these variables is very useful indicators to introduce tailored changes, on older people and their life contexts.

Therefore, the present study it was to enhance knowledge on the relationship between psychological variables such as social support, functionality and the spirituality and sociodemographic variables, and quality of life in elderly, in order to promote social support, empowerment on individual health and education policies adjust to the elderly people needs.

## Methods

### Study design and sample

This is a cross-section study performed in older people collect in the North of Portugal. After the authorization of the National Commission of Data Protection, the research team contacted several institutions of support to elderly in community in order to obtain authorization for the data collection. The data collection was also conducted in two Portuguese North Heath Institutions, in Private Hospital and University Clinic, after the Clinical Direction committee approved the study protocol.

Participants participated voluntarily, were informed about the study aims and signed an informed consent elaborated in accordance with the Declaration of Helsinki.

The data collection took place between March and September of 2017. The questionnaires were applied by research group in the institutions where the elderly people attended.

The sample was composed by 604 older people residents in the North of Portugal (Tâmega and Sousa Region), 63.6% were female gender with an average age of 71.6 (SD = 4.81) and with one to four years of schooling (92.7%). Most of the sample was married (67.2%), living with the marital partner (57%), included in a nuclear family (22.3%). Table 1 shows the sample characterization.

**Table 1:** *Sociodemographic and clinical characteristics of the sample*

Categorical variables	%(n)
**Gender (female)**	63.6(384)
**Marital Status**	
Single	6.6(40)
Married	67.2(406)
Divorced	1.2(7)
Widow	25.0(151)
**Education Level**	
Illiterate	4.6(28)
1 a 4 years	92.7(560)
5 a 12 years	2.5(14)
> 12 years	0.3(2)
**Co-habitation status**	
Partner	57.0(343)
Nuclear Family	22.3(134)
Extended family	3.2(19)
Institution / Senior Residence	0.8(5)
Alone	9.1(55)
Alone with assistance neighbour	1.5(9)
Alone with support of institution	3.2(19)
Alone without help	3.0(18)
**Clinical variables**	
Presence of chronic disease (yes)	72.0(435)
Prescription of chronic medication (yes)	87.1(526)
**Continuous variable**	**M (*SD*)**
**Age**	71.60(4.81)
**Min-Max**	65–94

Regarding the clinical characterization of the sample, most of the participants present chronic diseases (72%) and have prescription of chronic medication (87.1%). Analysing the results, there is a prevalence of the cardiovascular disease, diabetes and osteoarticular diseases and we are in the presence of a polymedicated population. In the context of this study, the sample includes people with rural habit and others with more urban habits and experiences. These elderly people, throughout their life course, include themselves in families with different economic situations, resulting from the transition from agricultural work to more industrialized work. Although the results were heterogeneous probably due to the low health literacy of the population.

#### Measures

*Sociodemographic questionnaire.* Participants completed background information about demographic (age, educational level, marital status, family characteristics) and clinical data (presence of chronic disease and medication).

The *Barthel Index* [25, 26] assess the degree of functionality and focuses on physical disability. It comprises 10 items rated on a Likert 4-points scale (0 to 3). Values ​​vary between 0 and 100 and a higher score indicates greater functionality. According to the Portuguese version, the cut off score of 60 indicate greater functionality. In this study, the alpha was .74.

*The Spiritual and Religious Attitudes in Dealing with Illness* [27, 28] assess how spirituality helps dealing with this chronic disease. It comprises 15 items rated on a Likert 5-points scale (1 to 5) and compromises three dimensions Search (for support/access), ii) Trust (in higher guidance/source) and iii) Reflection (positive interpretation of the disease) [16]. Values ​​vary between 15 and 75 and a higher score indicates greater spirituality to deal with chronic disease. In this study, the alpha was .95.

The *Satisfaction with Social Support Scale* [29] assess satisfaction with social support. It comprises 15 items rated on a Likert 5-point scale (1 to 5). Values ​​vary between 15 and 75 and a higher score indicates greater social support. In this study, the alpha was .86.

*Short Form Health Survey* (SF-36). This inventory assesses the quality of life [30] and the Portuguese version of Ferreira [31, 32] was used. This scale assesses two summary measures of quality of life: The Physical Quality of Life (PQL) and the Mental Quality of Life (MQL) and comprises eight scales of the concept Health Related Quality of Life (HRQoL): Physical functioning (PF), Role limitation due to physical health (RLP), Body pain problems (BP), General health perception (GH), Vitality (VT), Social functioning (SF), Role limitation due to emotional functioning (RE) and Mental health (MH). The first four scales reflect the perception of physical health status, and the following four the perception of psychological well-being. The instrument includes 11 items and 36 questions where each scale provides a sub score. The scores in each domain are transformed into measurements on scales of 0 to 100 and a higher score indicate greater HRQoL in PQL and MQL.

## Results

### Statistical analysis

Data were analysed using the IBM SPSS® software, version 24.0. In order to characterize the sample, descriptive statistics were performed through means and standard deviation for continuous variables and frequencies and percentages for nominal and categorical variables. Then, a correlation analysis was performed through a Pearson coefficient including age, spirituality, social support, functionality with MQL and PQL. A Point Bisserial was performed to test the relationships between gender and presence of chronic disease with MQL and PQL.

Path analysis was used to determine the pathways by which the psychological, sociodemographic and quality of life, interact to influence physical and mental quality of life. Path analysis allows obtaining direct and indirect effects between the variables and an indication of the overall suitability of the model. In the first model, gender was including in the model. However, as the model did not present a good fit, the variable was deleted from the model, and a second model was tested. In addition, spirituality was also not significantly associated with MQL or PQL. However, due to the high number of studies that show the influence of spirituality on quality of life, we have decided to include it, nonetheless. To determine the adequacy of the model Chi-Square Test fit value was examined taking into consideration the sample size [33]. As the significance of a chi-square test is dependent on the number of participants, other goodness-of-fit indexes were also used. Chi square/degree of freedom ratio (CMIN/DF) (<.5); goodness-of-fit statistic (GFI) and comparative fit index (CFI) and AGFI (> 0.95 suggesting good fit), and root mean square error of approximation (RMSEA) (> 0.05 suggesting good fit) [34, 35]. Based on multivariate Lagrange Multiplier (LM) tests, post-hoc modifications to the proposed model were made to add new paths as necessary. The significance of all direct and indirect effects was evaluated to determine which variables had a direct and indirect impact on mental and physical quality of life. Standardized beta coefficients (β) were derived for each explanatory variable to allow for the comparison and estimation of the relative importance of each measure. The R^2^ value was calculated for the outcome variables to determine the proportion of variance explained by the model [33]. Mediation analyses were evaluated by analysing the significance of the indirect effect between predictors and adjustment components by bootstrapping [36]. Significance level was set at 0.05. Path analysis was performed in IBM SPSS Amos® software, version 23.

### Descriptive statistics and relationships between variables

Pearson coefficients show that age, social support, and functionality were significantly associated with MQL and PQL. Point Bisserial show that the presence of chronic disease and gender were also significantly associated with MQL and PQL. Table 2 shows the descriptive statistics and the relationships between variables.

**Table 2:** Descriptive statistics and correlation coefficients (Pearson and Point Bisserial) for study variables

Variables	Mean (SD)	Range	2.	3.	4.	5.	6.	7.	8.
1.Age	71.60(4.81)	62–94	.039	−.014	−.014	−.187**	−.107**	−.212**	−.281**
2.Gender	–	–		.005	.163**	.011	−.159**	−.202**	−.191**
3.Presence of chronic disease	–	–			.042	−.042	−.111**	−.057	−.095*
4.Spirituality	47.76(11.34)	15–60				.019	.030	−.058	−.072
5. Functionality	96.12(13.12)	00–100					.270**	.337**	.386**
6.Social support	64.02(9.51)	15–75						.450**	.407**
7.Mental Quality of Life	52.94(11.03)	14–70							.801**
8.Physical Quality of Life	63.45(15.15)	21–87							

SD: standard deviation; **p < .01.

### Path analysis model

**Fig. 1:**
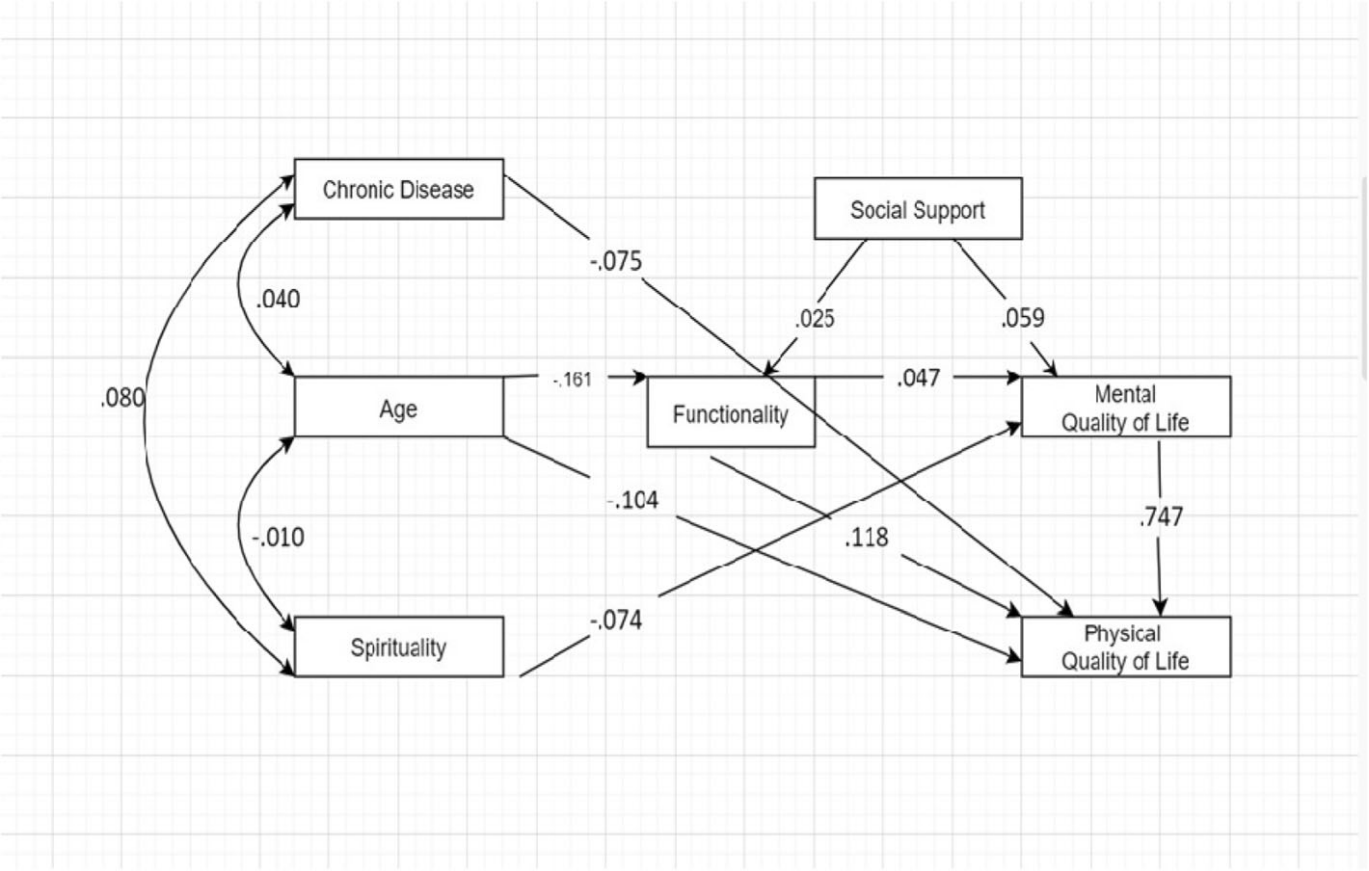
Path analysis with standardized direct effects. CI _95% =_ bias-corrected bootstrap confidence interval at 95% (1000 samples)

The multivariate linear regression final model for the mediation showed a good global adjustment: Fit indices: χ 2 (9) =39.6 (*p* < .001); χ 2 / df = 4.40; GFI = .982; AGFI = .944; CFI = .967; RMSEA = .075 (90% CI = (0.52; 1.00)). R-square indicates that this model (Fig. 1) can explain 26% of the variance in mental quality of life and 66% of the variance in physical quality of life. The final model shows that the presence of a chronic disease (β = −.075, *p* = .01) and age (β = −.104; *p* < .001) has a direct effect on physical quality of life. Spirituality is the only variable to have a direct effect on mental quality of life (β = −.074, *p* = .034). Also, age has a direct effect on functionality (β = −.161; p < .001), which in turn, has direct effect on physical quality of life (β = .118; p < .001). Mental quality of life has a direct effect in physical quality of life (β = .747; p < .001). Functionality has an indirect effect in mental quality of life being mediated by social support (β = .059; *p* < .01). Age has an indirect effect in mental quality of life being mediated by functionality (β = −.047; p < .01). (Table 3).

**Table 3:** *Standardized indirect effects for the mediation*

Variables			Indirect Effect	CI_**95%**_	
Predictor	Mediator	Outcome		Lower	Upper
Functionality	Social support	Mental quality of life	.059	.096	.258
Age	Functionality	Physical quality of life	−.047	−.018	−.078

** p < .01; CI_95%_ = Bootstrap bias-corrected confidence interval at 95% (1000 samples)

## Discussion

Although aging is considered above all a biological process, aging with quality of life is a biopsychosocial process. In fact, old age does not merely portray only a biological landmark. This is a life period characterized by marked social and psychological transformations, where various social and contextual factors interact: life conditions, family situation, support structures, personal expectations, among others [37]. In this context, aging and the way it is experienced by everyone is a process of great complexity, of individual experiences, deeply marked and influenced by external factors.

Therefore, the promotion of QoL in elders is a challenge for health and social care professionals. In order to understand the simultaneous relationship between modifiable (psychological variables) and non-modifiable variables (sociodemographic), and QoL in the elderly, a path analysis model was performed in order to guide and inform social, health and education policies.

As expected, individual characteristics showed a direct impact on physical and mental QoL. The presence of chronic disease had a negative impact on the PQL [38, 39] and is inversely associated with quality of life in the different domains: the elderly without any diseases present a significantly better quality of life [40] as well as the age of the individuals, i.e., older individuals had lower MQL. Being aging as a biological process this result makes an intuitive sense [41–43].

Interestingly, spirituality was the only variable to have a negative direct effect on MQL, emphasizing the role of spirituality on mental quality of life. In the face of aging, the difficulties inherent in the aging process and the inevitable sense of finitude, spirituality and religion are present as a support that helps the older people to counter the tendency to isolation and overcome the problems of daily life [23, 37, 39].

In this study, was evaluated how spirituality cope older people to deal with chronic disease, and, although most of this population reported having a chronic disease, however it is verified that their degree of autonomy is high. Thus, this chronic disease doesn’t cause or increase disability allowing the maintenance of the capacity to carry out their daily tasks. In fact, this older people don’t feel the need to search in God or in High power the support for adjust, find a meaning and understand her disease [9, 13, 23].

However, functionality showed an indirect effect in MQL being mediated by social support. Social support is a recognized mediator between functional status and QoL [16, 44, 45], being a resource to reduce adverse outcomes in older adults. Social support was a mediator between functional status and MQL emphasizing the importance to promote this resource in this population. Formal and informal groups are a way to enhance social participation, which has been pointed as an effective factor to improve the mental health of the older people [46]. Older adults who perceive a high social support show beneficial effects on enjoyment, morale, depression and loneliness [47].

Finally, in this model, the mental quality of life had a direct effect on physical quality of life of individuals. This result is interesting given that this relationship should be bidirectional but, in this sample, it seems that MQL affects PQL and not on the other way.

The results reinforce the need to address the individuals with chronic diseases, with a systematic and individualized social and health care in adapting policies to support healthy and active aging, designed for the national level, to the particular contexts of the lives of the elderly.

Physical activity should be promoted by institutions social network for the elderly as well as in the community such as churches and community gyms, in order to improve functionality and skills for daily activities due to the direct effect on PQL. Several studies have suggested that the level of physical activity recommended by WHO has a positive impact on QoL [48], especially with effects on general health, social function and mental health [49]. In addition, physical activity may protect against the cognitive decline characteristic of this population [50].

Finally, the fact that MQL affects PQL indicates that emotional well-being is an important topic to include in quality of life promotion programs for the elderly [51].. Portugal has a Strategy for Active and Healthy Development, 2017–2025, which defines the fundamental lines for a healthy and active lifestyle, focused, among other aspects, on the promotion of healthy lifestyles and health surveillance and on the creation safe environments, determinants for those who have more health and quality of life [52]. However, only through regional studies, as this one presents itself, carried out in a limited geographical territory, allowing a more in-depth knowledge of the internal and external factors that influence the quality of life.

Emotional support groups and leisure activities are healthy lifestyles that should be included in interventions programs [51]. Strategies such as reminiscence, theater, etc., can be productive in promoting the quality of mental life. In addition, social relations and leisure activities are associated with better health in the elderly [52]. These results highlight the importance of developing psychosocial intervention programs and at the level of health promotion that include strategies to develop functionality and social support in order to improve the quality of life of the elderly.

This study has some limitations that must be acknowledge. The questionnaire used to assess spirituality is specific for dealing with chronical diseases and not for using and dealing with everyday life, the loss and the aging process. The sample is limited to a geographical area where socio-cultural characteristics of urban and rural nature are mixed, that is, this area cannot be classified as entirely rural or urban, giving rise to limitations in extrapolating results to entirely urban or rural areas. The population surveyed, being representative of the Tâmega and Sousa region, is not representative of the country.

Other limitations stem from the extent of the survey applied. The data collection instrument used involved some extension, becoming difficult to apply and demanding attention to the respondents, which given the characteristics of low education of the population under study caused some difficulties in the interpretation of the questions.

However, this study represents an important contribution to the identification of the main social and clinical characteristics of the elderly population of a zone of the interior of Portugal that demands answers adapted to their needs.

## Conclusion

This study contributed for understand the variables who influence in quality of life of older people in the Tâmega and Sousa region in North of Portugal, that is a region where the incidence of chronic diseases is high. The finding showed that in elderly have chronic diseases reduce the physical and mental QoL, being more evidence this reduction in the older ones, as expected. Other fact is the importance and the role of social support in the increase of functionality and increase the quality o life. The social support mediated the relationship between the functionality and physical quality of life reinforce the importance to developed psychosocial and heath programs in region that include physical activity, literacy in heath and a social active life. These results will contribute, in the future, to the development of psychosocial guidelines and in the social network to that improve the quality of life of the elderly.

The results of this study point out a specific need to improve knowledge related with skills that make possible to understand the disease symptoms and how to deal with them. The strategies should include programs related with health education with the main point: self-management symptoms and heath literacy. At the same time in each community it may be a useful strategy to introduce specific program where elderly can share their life experience related with their last occupation to perpetuate culture social ties. Also, a tailored approached related with the potentiality of digital approaches must be important to minimize the loneliness and isolation.

It would be a good policy that the programs related with strategies to improve life quality and well- being of the elderly be planned with different community resources, to see the needs of the elderly globally and develop different approaches together, as a continuing path. This seems to be more efficient and less expensive with gains to the elderly, families and communities.

## Abbreviations

BP: Body pain problems
CMIN/DF: Chi square/degree of freedom ratio
CFI: Comparative fit index
GH: General health perception
GFI: Goodness-of-fit statistic
HRQoL: Health Related Quality of Life
LM: Lagrange Multiplier
MQL: Mental Quality of Life
MH: Mental health
PQL: Physical Quality of Life
PF: Physical functioning
RE: Role limitation due to emotional functioning
RLP: Role limitation due to physical health
SSSS: Satisfaction with Social Support Scale
SF-36: Short Form Health Survey
SF: Social functioning
SpREUK: Spiritual and Religious Attitudes in Dealing with Illness
VT: Vitality
